# A Novel Cyclodextrin Glycosyltransferase from Alkaliphilic *Amphibacillus* sp. NPST-10: Purification and Properties

**DOI:** 10.3390/ijms130810505

**Published:** 2012-08-22

**Authors:** Abdelnasser S. S. Ibrahim, Ali A. Al-Salamah, Mohamed A. El-Tayeb, Yahya B. El-Badawi, Garabed Antranikian

**Affiliations:** 1 Department of Botany and Microbiology, College of Science, King Saud University, Riyadh 11451, Kingdom of Saudi Arabia; E-Mails: aaasalah@maktoob.com (A.A.A.-S.); mali5@ksu.edu.sa (M.A.E.-T.); yelbadawi@ksu.edu.sa (Y.B.E.-B.); 2 Institute of Technical Microbiology, Hamburg University of Technology, Kasernenstrasse 12, 21073 Hamburg, Germany; E-Mail: antranikian@tuhh.de

**Keywords:** alkaliphiles, soda lakes, cyclodextrin glycosyltransferase, *Amphibacillus* sp., purification, 16S rDNA

## Abstract

Screening for cyclodextrin glycosyltransferase (CGTase)-producing alkaliphilic bacteria from samples collected from hyper saline soda lakes (Wadi Natrun Valley, Egypt), resulted in isolation of potent CGTase producing alkaliphilic bacterium, termed NPST-10. 16S rDNA sequence analysis identified the isolate as *Amphibacillus* sp. CGTase was purified to homogeneity up to 22.1 fold by starch adsorption and anion exchange chromatography with a yield of 44.7%. The purified enzyme was a monomeric protein with an estimated molecular weight of 92 kDa using SDS-PAGE. Catalytic activities of the enzyme were found to be 88.8 U mg^−1^ protein, 20.0 U mg^−1^ protein and 11.0 U mg^−1^ protein for cyclization, coupling and hydrolytic activities, respectively. The enzyme was stable over a wide pH range from pH 5.0 to 11.0, with a maximal activity at pH 8.0. CGTase exhibited activity over a wide temperature range from 45 °C to 70 °C, with maximal activity at 50 °C and was stable at 30 °C to 55 °C for at least 1 h. Thermal stability of the purified enzyme could be significantly improved in the presence of CaCl_2_. *K*_m_ and *V*_max_ values were estimated using soluble starch as a substrate to be 1.7 ± 0.15 mg/mL and 100 ± 2.0 μmol/min, respectively. CGTase was significantly inhibited in the presence of Co^2+^, Zn^2+^, Cu^2+^, Hg^2+^, Ba^2+^, Cd^2+^, and 2-mercaptoethanol. To the best of our knowledge, this is the first report of CGTase production by *Amphibacillus* sp. The achieved high conversion of insoluble raw corn starch into cyclodextrins (67.2%) with production of mainly β-CD (86.4%), makes *Amphibacillus* sp. NPST-10 desirable for the cyclodextrin production industry.

## 1. Introduction

Cyclodextrin glycosyl transferase (CGTase, EC 2.4.1.19) is an important industrial enzyme, unique in its ability to convert starch and related glycans into non-reducing, cyclic malto-oligosacchrarides called cyclodextrins (CDs) via a cyclization reaction, an intramolecular transglycosylation reaction [1]. Moreover, it is an important hydrolytic enzyme that carries out reversible intermolecular coupling and disproportionation of maltooligosaccharides [1,2]. CDs are non-reducing cyclic structures consisting mainly of 6, 7 or 8 glucose residues, joined by α-(1,4) linkages, for α-, β- and γ CD cyclodextrin, respectively. The arrangement of glucose units in the CD molecule results in the shape of a hollow truncated cone with a hydrophilic external surface, which makes CDs water-soluble, and a hydrophobic internal cavity, which enables CDs to accommodate and form inclusion complexes with various organic and inorganic compounds [3,4]. The profound advantageous effects on the guest molecules after formation of inclusion complexes with cyclodextrins, has led to many applications of cyclodextrins in various areas including textile, pharmaceuticals, bioconversion, cosmetics, environmental protection, and food industries [5,6].

Most CGTases convert starch into a mixture of α-, β- and γ-CD in different ratios, and depending on the main cyclodextrin produced, CGTases are classified as α-, β- or γ-CGTases [7]. Among the three types of cyclodextrins, β-CD is of high interest due to the size of its non-polar cavity which is suitable to encapsulate several guest molecules; its low solubility in water which facilitates its separation from the reaction mixture. Moreover, β-CD inclusion complexes are easily prepared and more stable [6,8]. As the separation of different CDs is costly and time-consuming, CGTases that synthesize predominantly one type of CD are of great interest.

Alkaliphilic microorganisms have attracted much interest in the past few decades because of their ability to produce extracellular enzymes that are active and stable at high pH values [9,10]. The main natural habitats of alkaliphiles are alkaline environments. Naturally occurring alkaline environments, such as carbonate springs, alkaline soils, and soda lakes, are characterized by their high basic pH values (pH 8.0–11.0) due to the presence of high concentrations of sodium carbonate salts formed by evaporative concentration [11,12]. Soda lakes are widely distributed all over the world; however, as a result of their inaccessibility, few have been explored from a microbiological point of view [13]. The Egyptian hyper saline soda lakes in the Wadi Natrun area (30°15′N, 30°30′E) are an excellent example of these hitherto unexplored alkaline ecosystems. In this work, we present isolation of novel CGTase producing alkaliphilic bacterium from hyper saline soda lakes, purification and characterization of the CGTase, in addition to some studies on cyclodextrins production by the purified enzyme.

## 2. Results and Discussion

### 2.1. Isolation of CGTase Producing Alkaliphilic Bacteria

Screening of alkaline water and sediment samples collected from Wadi Natrun hyper saline soda lakes for isolation of CGTase producing alkaliphilic bacteria, using rich alkaline agar medium containing 0.02% (*w*/*v*) phenolphthalein, resulted in isolation of a few isolates showing halo zones around their margins, suggesting possibility of CGTase production by those strains [14]. The positive isolates were propagated in alkaline liquid medium and CGTase activity was measured in the cultures supernatants. One of those isolates termed NPST-10, showing the highest CGTase production (0.4 U/mL), was selected for further study. In order to determine the phylogenetic position of strain NPST-10, 16S rDNA analysis was performed. A total of 870 nucleotides of NPST-10 16S rRNA gene were determined corresponding to 661 to 1531 of *E. coli* numbering. According to the phylogenetic analysis, NPST-10 is a member of the genus *Amphibacillus* with similarity of 98%. Hence, it was designated as *Amphibacillus* sp. strain NPST-10. The 16S rDNA gene sequence was deposited in the GeneBank under Accession No. JX028596. The genus *Amphibacillus* was first proposed in 1990 by Niimura *et al*. [15]. The genus currently comprises four recognized species including *Amphibacillus xylanus* [15], *Amphibacillus fermentum*, *Amphibacillus tropicus* [16], and *Amphibacillus sediminis* [17]. To the best of our knowledge this is the first report of CGTase production by *Amphibacillus* sp.

### 2.2. Purification of the CGTase

**Figure 1:**
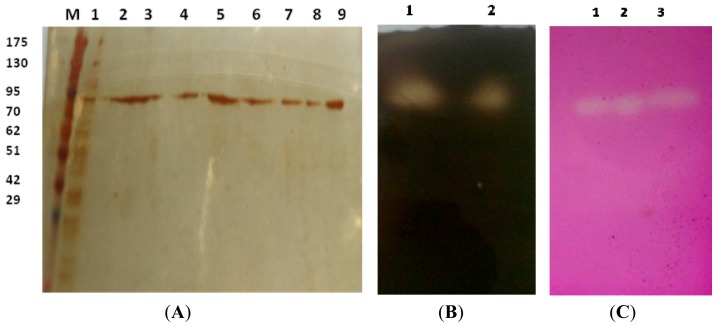
(**A**) SDS-PAGE analysis of purification steps of *Amphibacillus* sp. NPST-10 CGTase on 7.5% SDS-polyacrylamide. M, Protein marker (GeneDirex); lane 1: Crude enzyme (culture supernatant); lane 2 and 3: Concentrated corn starch eluate (50×); lane 4–9: DEAE-Cellulose fractions. Protein bands were detected by silver staining. Native PAGE; (**B**) Starch degrading activity staining. lane 1: Crude enzyme (supernatant); lane 2: Corn starch eluate; (**C**) CGTase activity staining. lane 1: Crude enzymes (supernatant); lane 2 and 3: Corn starch eluate.

CGTase from *Amphibacillus* sp. NPST-10 was purified by adsorption of the crude enzyme to corn starch followed by elution of the enzyme using 1 mM β-CD solution. Further purification step was performed by application of the concentarted eluate to DEAE-cellulose column for separation of any minute impurities. However, single protein band obtained in SDS-PAGE indicated the purity and homogeneity of the purified CGTase, was achieved using only corn starch adsorption procedures (Figure 1A). CGTase was purified up to 22.1 fold with recovery of 44.7%, and the result of purification steps are summarized in Table 1. A fast specific CGTase activity staining, rather than the non-specific starch/iodine staining, was developed and optimized as described in the materials and methods section. CGTase activity was detected in both the crude enzyme (culture filtrate) and the purified CGTase with the same molecular weight (Figure 1B,C). Different separation procedures have been previously applied for obtaining purified CGTases, and in most cases, three or four purification steps were applied including ultrafiltration, gel filtration, starch adsorption and ion exchange chromatography [2,18–21] or ammonium sulfate precipitation and two steps ion exchange chromatography [22–24].

In addition to cylization activity of the purified *Amphibacillus* sp. NPST-10 CGTase, coupling and hydrolytic activities were determined. Catalytic activities of the enzyme were found to be 88.8 U mg^−1^ protein, 20.0 U mg^−1^ protein and 11.0 U mg^−1^ protein, for cyclization, coupling and hydrolytic activities, with ratio of 1:0.23:0.12, respectively. The coupling/cyclization activity ratio of *Amphibacillus* sp. NPST-10 CGTase (0.23:1.0) was less than that recently reported for *Bacillus sphaericus* strain 41, (0.3:1.0), respectively [3]. Depending on the source organism, CGTases often display varying levels of coupling, disproportionation, and hydrolytic activities besides that of cyclization [3,25]. Although these reactions all involve the same catalytic residues, the chemical mechanism of catalysis by CGTase is a double-displacement reaction involving a covalent enzyme-intermediate complex. Nevertheless, the transglycosylation reaction was found to proceed via different kinetic mechanisms [3,26].

### 2.3. Properties of *Amphibacillus* sp. NPST-10 CGTase

#### 2.3.1. Estimation of Molecular Weight

The molecular weight of the purified denatured CGTase was estimated to be 92 kDa (Figure 1A) by SDS-PAGE. The purified native and denatured enzymes showed single band with similar molecular weight on native PAGE and SDS-PAGE, respectively, suggesting that NPST-10 CGTase is a monomeric protein. Most of the reported CGTases are monomeric in nature with molecular weight between 60 and 110 kDa [23,27–29]. However, CGTases with lower molecular weight have been also reported, such as 33 kDa from *Bacillus coagulans* [30] and as 56 kDa from *Bacillus sphaericus* strain 41 [3].

#### 2.3.2. Effect of Temperature on CGTase Activity and Stability

**Figure 2:**
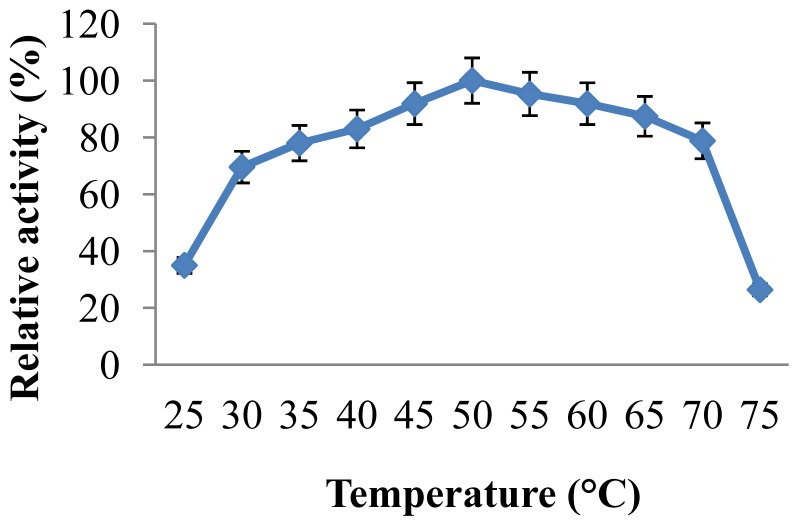
Effect of temperature on CGTase activity of *Amphibacillus* sp. NPST-10. CGTase activity was measured under the standard assay conditions at various temperatures (25 °C–75 °C) at pH 8. Results represent the mean of three separate experiments, and error bars are indicated.

**Figure 3:**
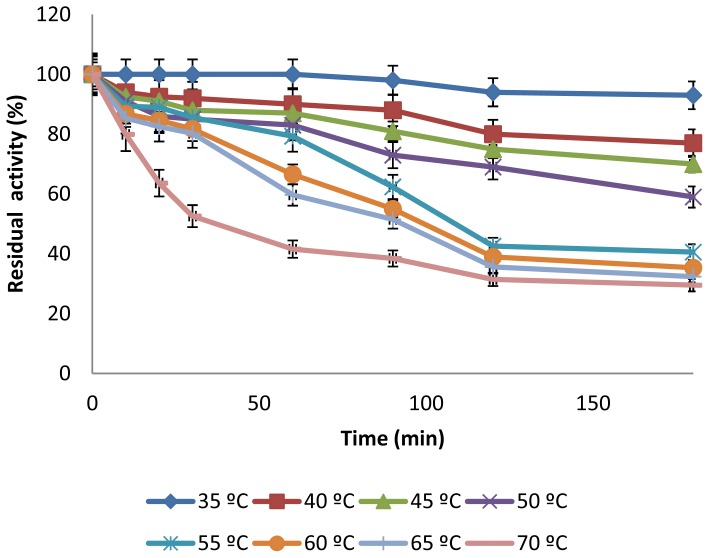
Thermostability of *Amphibacillus* sp. NPST-10 CGTase. The purified enzyme in Tris-HCl buffer (pH 8.0) was pre-incubated at different temperatures (35 °C–70 °C) for different time intervals (10–180 min) before measurement of residual activity under the standard assay conditions. Results represent the mean of three separate experiments, and error bars are indicated.

The temperature profile of the enzyme was estimated by measurement of the enzyme activity at various temperatures (Figure 2). CGTase showed significant activity in a wide temperatures range, 45 °C–70 °C, showing maximal enzyme activity at 50 °C. The relative enzyme activities at 60, 65 and 70 °C were found to be 92%, 87.4% and 78.8%, respectively. However, increasing of the reaction temperature to 75 °C caused rapid decrease of the relative enzyme activity to 26.4%. Temperature optima in the range of 55–65 °C have been previously reported for CGTases from various alkaliphiles [20,23,27,29,31]. However, CGTase from thermophilic bacteria usually shows optimal activity at higher temperatures [28,32]. In addition, the thermal stability of the purified CGTase was investigated by pre-incubation of the enzyme at different temperatures at pH 8.0 for different time intervals before measurement of residual activities. The results illustrated in Figure 3, showed that the stability of the enzyme depended on the temperature and period of preheating. At 30 °C to 55 °C, the enzyme retained between 80% to 100% of its initial activity after enzyme treatment for 1 h, and retained 40–93% to 40% after 3 h of treatment. Moreover, CGTase retained 81.3%, 80.3% and 52.6% of the initial activity after treatment for 1 h at 60, 65 and 70 °C, respectively. More adverse effect was found by treating the enzyme for 3 h at 60–70 °C, at which the enzyme retained only 30% to 35% of its initial activity.

**Figure 4:**
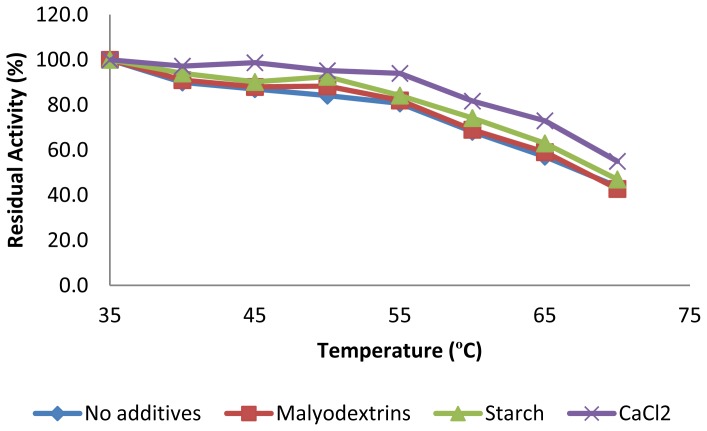
Effect of CaCl_2_, starch and maltodextrin on the thermostability of the purified CGTase from *Amphibacillus* sp NPST-10. The enzyme was pre-incubated in the presence of calcium salt (1 mM), starch (1%, *w*/*v*), or maltodextrin (1%, *w*/*v*), respectively, at varying temperatures range (35 °C–70 °C) for 1 h prior to determination of the residual enzyme activity under the standard assay conditions. Standard deviations of the relative activities were in the range of 1.5%–3.6%.

Earlier reports have shown that CGTase thermostability is enhanced in the presence of its substrate, products, and calcium ions [20,29,32]. Therefore, the influence of these additives on the thermostability of *Amphibacillus* sp. NPST-10 CGTase was studied. It was found that the thermal stability of CGTase was enhanced by about 1.3 fold in the presence of calcium ion, and slightly enhanced in the presence of maltodextrins or starch (Figure 4). Structural analysis of the CGTases have shown the presence of two calcium ions, one near the *N*-terminal end and the other near the active site region, provide stability to the conformational structure of flexible regions of the protein molecule including the active site [28].

#### 2.3.3. Effect of pH on CGTase Activity and Stability

**Figure 5:**
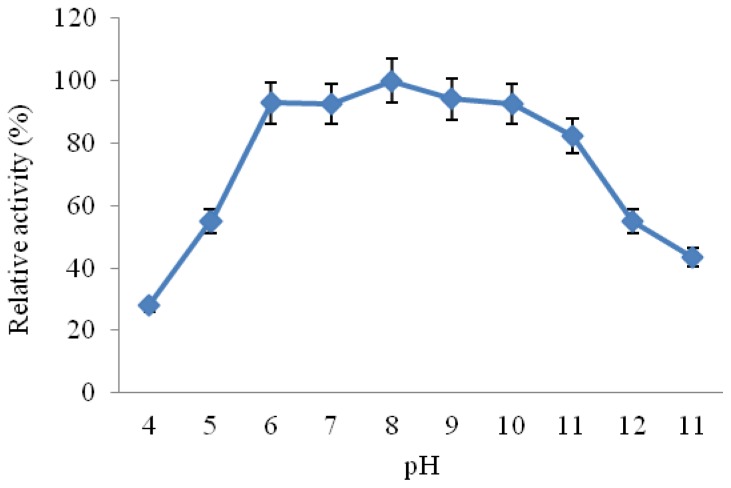
Effect of pH on the activity of *Amphibacillus* sp. NPST-10 CGTase. The enzyme activity was measured under the standard assay conditions at various pH values at 50 °C. Results represent the mean of three separate experiments, and error bars are indicated.

The influence of pH on the CGTase activity was established by measurement of enzyme activity at varying pH values ranging from 4.0 to 11.0 at 50 °C under standard assay conditions. The results shown in Figure 5 indicated that the enzyme was active in a wide pH range from 6.0 to 9.0 with relative activities of 75% to 85%, showing maximal activity at pH 8. It has been reported that CGTases from alkaliphiles showed optimal cyclizing activities in pH range from 5.0 to 10.0 depending on the bacterial producer [3,20,27]. However, some CGTases exhibited two pH peaks at pH 6.0 and 9.0, such as CGTase from *Bacillus* sp. [7–12,23], *Bacillus firmus* [32], and *Bacillus pseudalcaliphilus* 20RF [29].

**Figure 6:**
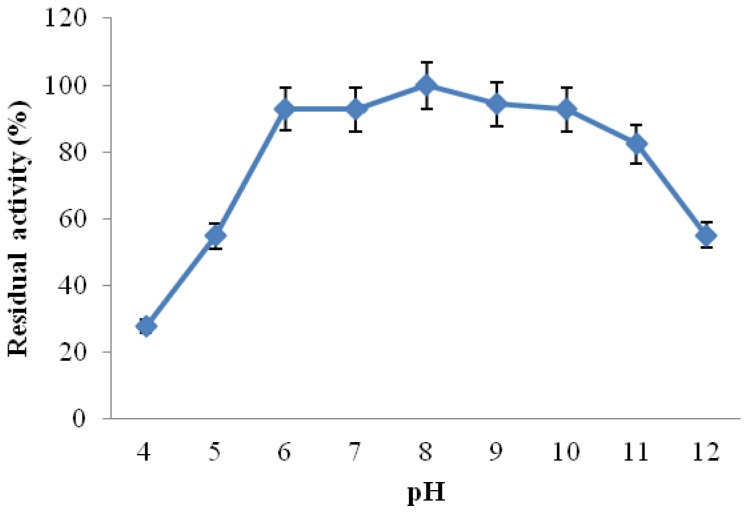
Effect of pH on the stability of *Amphibacillus* sp. NPST-10 CGTase. The enzyme was pre-incubated in buffers of various pH values for 1 h at 25 °C, and then the residual activities were measured under the standard assay conditions. Results represent the mean of three separate experiments, and error bars are indicated.

Investigation of the pH stability of *Amphibacillus* sp. NPST-10 CGTase was performed by pre-incubation of the enzyme in buffers of various pH values for 1 h at 25 °C prior to determination of the residual activities under the standard assay conditions. The enzyme retained about 93%–100% of its initial activity in a wide range of pH values, between pH 6.0 and 10.0 (Figure 6). Moreover, the enzyme retained more than 82% of its initial activity at pH 11.0, but was less stable in pH 5.0 and 12.0, showing residual activity of 55% and 49.2%, respectively. In comparison to other CGTases reported from alkaliphiles, *Amphibacillus* sp. NPST-10 enzyme showed high stability in wide pH range, slightly higher than alkaliphilic *Bacillus pseudalcaliphilus* 20RF CGTase [29]. Moreover, *Amphibacillus* sp. NPST-10 CGTase was much more stable than CGTase from alkaliphilic *Bacillus sphaericus* strain 41, where at pH 5.0, the activity retained was 34.2%, while in the range of pH 8.0–10.0 the average retention of activity was 16.8% [3], indicating that *Amphibacillus* sp. NPST-10 CGTase could be successfully applied for prodyction of cyclodextrins in range of pH from 5.0 to 11.0 for production of cyclodextrins.

#### 2.3.4. Effect of Various Reagents and Metal Ions on CGTase Activity

In order to be establish the effect of various metal ions and reagents on CGTase activity, enzyme assays were carried out in presence of theses additives under the standard assay conditions. A significant lose of the enzyme activity was established in the presence of 10 mM Co^2+^, Zn^2+^, Cu^2+^, Cd^2+^ or Hg^2+^ with residual activities of 82.1%, 40.3%, 32.8% and 29.3% and 0.0%, respectively (Table 2). These results are similar to those reported by Atanasova *et al*. [29], significant decrease of CGTase activity of alkaliphilic *Bacillus pseudalcaliphilus* 20RF in the presence of 15 mM Zn^2+^ and Co^2+^ ions. However, the inhibitory effect of Co^2+^ on *Amphibacillus* sp. NPST-10 CGTase is in contrast to that reported by Martins and Hatti-Kaul [33], where cobalt had no effect on the activity of CGTase from *Bacillus agaradhaerens* LS-3C. In addition, Li *et al*. [21] has recently reported activation of CGTase from *Paenibacillus macerans* by Zn ion. CGTase of *Amphibacillus* sp. NPST-10 was slightly inhibited in the presence of manganese and magnesium ions. Ba^2+^ ion was found to significantly inhibit CGTase of *Amphibacillus* sp. NPST-10, which is similar to that reported for CGTase from *B. agaradhaerens* LS-3C [33], but in contrast to that reported by Li *et al*. [21] where Ba^2+^ has stimulatory effect on CGTase from *Paenibacillus macerans*. The effect of metal ions and reagents on the activity of CGTases depends on the bacterial producer. However, the inhibitory effect of ions is mostly attributed to the metal catalyzed oxidation of amino acid residues essential to the enzyme activity [29]. The most probable candidates would be tryptophan which is known to be present in the active site and specific tyrosine and histidine residues which are ascribed an important role in cyclization efficiency and in transition state stabilization [1]. The reducing agent, 2-mercaptoethanol, resulted in significant inhibition of *Amphibacillus* sp. NPST-10. EDTA, a metal chelating agent, had no effect on the enzyme activity, suggesting that this CGTase is not a metalloenzyme, which is similar to that reported for CGTase from alkaliphilic *Bacillus pseudalcaliphilus* 20RF [29]. *Amphibacillus* sp. NPST-10 CGTase activity was slightly stimulated in the presence of Ca^2+^, which appeared to be a characteristic feature of different bacterial CGTases [27,34].

Among the CDs, 1 mM of β- and γ-CD showed significant inhibitory effect on the enzyme activity with residual activity of 45.7% and 54.2%, respectively. Some authors have reported total inhibition of CGTase by the reaction products. For instance, CGTase from *B. megaterium* showed a total loss of activity in the presence of 12 mg/mL of β-CD [35], as did the CGTase from *B. agaradhaerens* LS-3C and *Bacillus circulans* DF 9R [33,36].

#### 2.3.5. Kinetic Parameters

**Figure 7:**
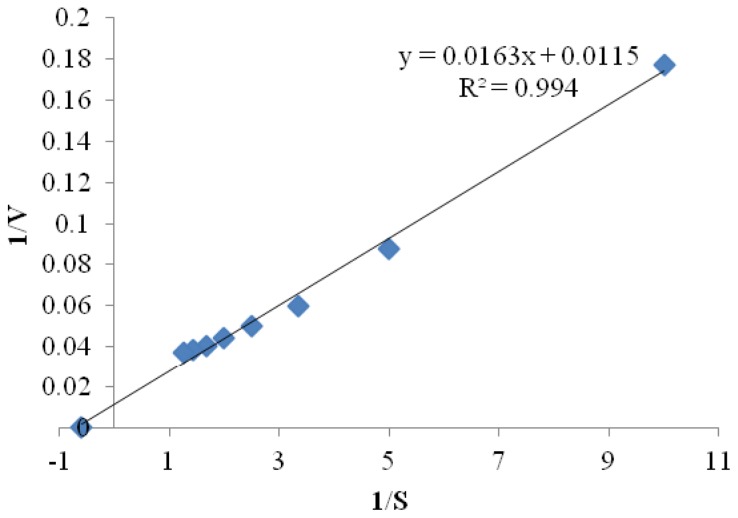
Estimation of kinetic constants of *Amphibacillus* sp. NPST-10 CGTase. The enzyme activity was measured at various starch concentrations (0.1–1.5 mg/mL) at pH 8.0 and 50 °C. *K*_m_ and *V*_max_ constants were determined using linearized Lineweaver-Burk plot.

Kinetic studies of the purified CGTase were investigated by measuring initial rates of CGTase reaction at different concentrations of soluble starch in 50 mM Tris-HCl buffer (pH 8.0) at 50 °C. The kinetic parameters, *K*_m_ and *V*_max_, were estimated using Michaelis-Menten equation and double reciprocal plot known as Lineweaver-Burk plot [33]. The *K*_m_ and *V*_max_ values using soluble starch as a substrate were estimated to be 1.7 ± 0.15 mg/mL and 100 ± 2.0 μmol/min, respectively (Figure 7). The low value of *K*_m_ indicates the high affinity of *Amphibacillus* sp. NPST-10 CGTase toward the substrate. *K*_m_ values ranging from 1.77–5.7 mg/mL and *V*_max_ from 43–1027 μmol/min have been previously reported for various CGTases [21–24,37].

#### 2.3.6. Substrate Specificity

**Figure 8:**
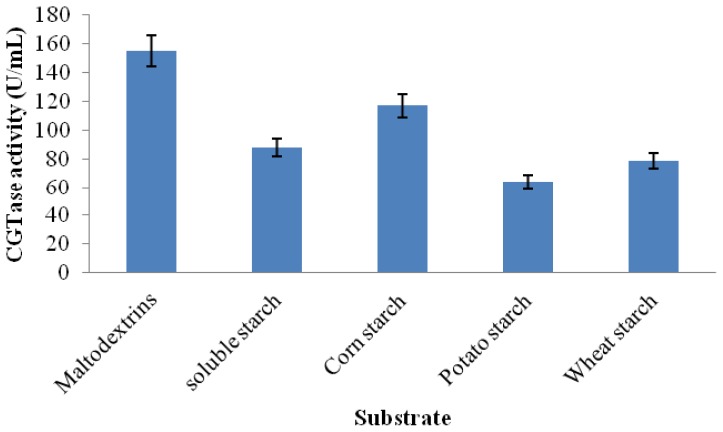
Activity *Amphibacillus* sp NPST-10 CGTase using various substrates. Results represent the mean of three separate experiments, and error bars are indicated.

The sources of starch could affect the CGTase activity, which is probably due to the differences in the starch granules structure and properties among various starch sources [37]. Hence, the action of the purified CGTase on hydrolyzed and raw substrates was studied. The results presented in Figure 8 indicated that the enzyme possesses activity on all of the tested substrates, whether partially hydrolyzed (soluble starch) or raw starch. Interestingly, *Amphibacillus* sp. NPST-10 CGTase showed higher activity using raw cornstarch than soluble starch (partially hydrolyzed starch). However, the cyclization activity using maltodextrin, short oligosaccharides (17 glucose residues) was the highest. The raw starch has a compact crystalline structure that is not easily degraded, and therefore CGTases with high activity toward raw starch is highly recommended for industrial production of CDs [31].

#### 2.3.7. Production of Cyclodextrins

The production of CDs was analyzed by HPLC using pure CGTase and 1% (*w*/*v*) of soluble starch as a substrate, at 50 °C and pH 8.0. A maximum yield of CDs with about 67.2% conversion of soluble starch into CDs could be obtained after 4 h and the products ratio at that point was 10.2% α-CD, 86.4% β-CD and 3.4% γ-CD. The production yield and ratio of the different CDs formed by CGTases are dependent not only on the microbial source producing the enzyme, but also on the nature of the substrate and reaction conditions, such as temperature, pH and reaction time [5]. Depending on the most abundant form of cyclodextrin that CGTase produces, the enzyme is sometime classified as α-, β- and γ-CGTase [1,25,37]. By this classification, *Amphibacillus* sp. NPST-10 CGTase could be considered as β-CGTase. Of the three kinds of CDs, β-CD is of most practical use because its inclusion complexes are easily prepared and more stable. The size of the β-CD polar cavity is optimum for many molecules such as drugs and preservatives. Furthermore, β-CD is easily separated from the reaction mixtures due to its low solubility in water [38].

## 3. Materials and Methods

### 3.1. Collection of Soil and Water Samples

Sediment and water samples were collected from hyper saline soda lakes in Wadi Natrun valley located in northern Egypt. Wadi Natrun valley extends in a northwest by southeast direction between latitudes 30°15′ north and longitude 30°30′ east. The bottom of Wadi Natrun valley is 23 m and 38 m below sea level and water level of Rosetta branch of the Nile, respectively [39]. Sediment and water samples were collected from various hyper saline soda lakes in sterile containers and kept at 4 °C.

### 3.2. Isolation of CGTase Producing Alkaliphilic Bacteria

Isolation of CGTase-producing alkaliphilic bacteria was carried out using rich alkaline agar medium containing 0.02% (*w*/*v*) phenolphthalein, as an indicator of cyclodextrin production [14]. The alkaline agar medium (pH 10.5) contained soluble starch (10 g/L), yeast extract (5 g/L), casamino acids (5 g/L), peptone (5 g/L), NaCl (50 g/L), Na_2_CO_3_ (15 g/L), agar (15 g/L) and 300 μL trace elements solution. The trace elements solution contained: CaCl_2_·2H_2_O (1.7 g/L), FeSO_4_·7H_2_O (1.3 g/L), MnCl_2_·4H_2_O (15.4 g/L), ZnSO_4_·7H_2_O (0.25 g/L), H_3_BO_3_ (2.5 g/L), CuSO_4_·5H_2_O (0.125 g/L), Na_2_MoO_4_ (0.125 g/L), Co(NO_3_)_2_·6H_2_O (0.23 g/L) and 2.5 mL 95%–97% H_2_SO_4_. Na_2_CO_3_ and trace elements solutions were autoclaved separately before addition to the medium. Sediment and water samples were suspended and serially diluted in a 10% (*w*/*v*) NaCl solution prepared in 50 mM glycine-NaOH buffer, pH 10. Aliquots (200 μL) of different dilutions were spread on the alkaline agar medium and incubated at different temperatures, for several days. Formation of halo zone around the colonies, resulted from the production of phenolphthalein-cyclodextrin inclusion complexes, was considered as an initial indication of CGTase activity [14].

### 3.3. Bacterial Identification

The selected strain was identified using 16S rRNA gene sequence analysis. The bacterial isolate was grown overnight in 5 mL alkaline broth medium. Total DNA was extracted using DNeasy Blood & Tissue Kits (Qiagen, NY, USA) according to the manufacturer’s instructions. Eubacterial-specific forward primer: 16F27 (5′-AGA GTT TGA TCC TGG CTC AG-3′), and reverse primer: 16R1525 (5′-AAG GAG GTG ATC CAG CCG CA-3′) were used to amplify 16S rDNA gene [29]. PCR amplification was performed in a final reaction volume of 50 μL and the reaction mixture contained 25 μL GoTaq^®^ Green Master Mix (2×), (Promega, USA), 1 μL forward primer (10 μM), 1 μL reverse primer (10 μM), 5 μL DNA template (200 ng) and 18 μL nuclease-free water. The PCR reaction was run for 35 cycles in a DNA thermal cycler under the following thermal profile: Initial denaturation at 95 °C for 5 min, denaturation at 95 °C for 1 min, primers annealing at 52 °C for 1 min and extension at 72 °C for 1.5 min. The final cycle included extension for 10 min at 72 °C to ensure full extension of the products. PCR products were run in agarose gel electrophoresis and purified using a QIAquick gel extraction kit (Qiagen, USA), and sequenced using an automated sequencer (Research center, King Faisal Hospital, Riyadh, Saudi Arabia). The obtained 16S-rDNA gene sequence of the isolate was aligned with reference 16S-rDNA sequences of the European Microbiological Laboratory (EMBL), GenBank (gb, Germany) and the data base of Japan (dbj) using the BLAST algorithm available in NCBI homepage (National Centre for Biotechnology information) [40].

### 3.4. CGTase Production and Purification

Colonies of the selected strain were transferred to 250 mL Erlenmeyer flasks containing 50 mL of alkaline liquid culture medium with the same composition as the solid medium, except for the presence of agar and dye, and incubated for overnight at 30 °C under orbital shaking (120 rpm). This culture was used to inoculate (2%) one liter Erlenmeyer shaking flask containing 250 mL of the same medium and cultivated under the same conditions for approximately 48 h. Cells and insoluble materials were removed by centrifugation at 6000*g* for 15 min at 4 °C, and cell-free supernatant was filtered through a 0.45-μm pore-size membrane filter and used as source of crude CGTase.

CGTase was purified using two steps including corn starch adsorption and ion exchange chromatography. Insoluble corn starch and ammonium sulfate were added to one liter of cell-free supernatant to concentrations of 5% (*w*/*v*) and 1 M, respectively, and kept at 4 °C with continuous gentle agitation for 1 h to allow enzyme adsorption. The mixture was then centrifuged at 5000*g* for 10 min and the pellet was washed twice with cold ammonium sulphate solution (1 M) to remove any unbound proteins. In order to eluate the adsorbed CGTase from the corn starch, the residue was incubated with 200 mL of 50 mM Tris-HCl buffer, pH 8.0, containing 1 mM β-CD, for 30 min at 37 °C with shaking followed by centrifugation to give eluate 1. The elution was repeated with 80 mL of the same β-CD solution to give eluate 2. The eluates (1 and 2) were pooled (280 mL), dialysed against 50 mM Tris-HCl buffer (pH 8.0) at 4 °C. This eluate was lyophilized using freeze dryer and the solid materials were resuspended in 5 mL of 50 mM Tris-HCl buffer (pH 8). The sample was centrifuged and filtered through a 0.45-μm pore-size membrane filter. The concentrated sample was applied to glass column (1.5 × 20 cm) containing DEAE-cellulose, pre-equilibrated with 50 mM Tris-HCl buffer (pH 8). The column was washed with the same buffer and proteins were eluted using a linear 0–1 M NaCl gradient in the same buffer at flow rate of 1.5 mL/min. Fractions (2 mL) were collected and absorbance was monitored at 280 nm. Fractions containing CGTase activity were pooled and dialyzed against 50 mM Tris-HCl buffer overnight at 4 °C. All purification steps were performed in cold room.

### 3.5. Enzyme Assays

#### 3.5.1. Cyclization Activity

Cyclization activity of CGTase was measured as β-CD forming activity according to a method previously described with some modification [33]. Seven hundred and fifty micro liter of 1% (*w*/*v*) starch solution prepared in 50 mM Tris-HCl buffer, pH 8 was pre-incubated at 50 °C for 5 min. One hundred micro liter of enzyme sample was added to the reaction mixture and after incubating for 20 min at 50 °C, the reaction was quenched by adding 375 μL of 0.15 M NaOH. Subsequently, 100 μL of 0.02% (*w*/*v*) phenolphthalein prepared in 5 mM Na_2_CO_3_ was added, and after standing at room temperature for 15 min, the color intensity was measured at 550 nm. One unit of CGTase activity was defined as the amount of enzyme releasing 1 μmol of β-CD per min under the defined assay conditions. A calibration curve was made using 0.001–0.5 μmol of β-CD in 50 mM Tris-HCl (pH 8). Protein concentration was determined according to the method described by Bradford [41] with bovine serum albumin as the standard protein.

#### 3.5.2. Hydrolytic Activity

Hydrolytic activity of CGTase was performed by incubation of 750 μL 1% starch solution, prepared in 50 mM Tris-HCl buffer (pH 8), for 30 min at 50 °C. To terminate the enzymatic reactions, the mixtures were boiled for 5 min. The release of reducing sugars was followed by dinitrosalicylic acid method [42]. 0.1 mL of the reaction mixture was added to 1 mL of 3,5-dinitrosalycilic acid reagent and incubated in boiling water bath for 10 min, cooled and the absorbance was measured at 546 nm. One unit of hydrolytic activity of CGTase was defined as the amount of enzyme releasing 1 μmol of reducing sugars per minute under the defined assay conditions. A calibration curve was made using 0.1–1 mg/mL d-glucose.

#### 3.5.3. Coupling Activity

Coupling activity of the purified CGTase was measured according to the disappearance of β-CD in the presence of glucose using modification a protocol described previously [3]. Enzyme solution (100 μL) was added to 750 μL of 50 mM Tris-HCl buffer containing 0.5 mM β-CD and 1% glucose starch solution prepared in 50 mM Tris-HCl buffer (pH 8) and incubated for 30 min at 50 °C. The reaction was stopped by boiling for 5 min and the amount of residual β-CD was determined calorimetrically as described in Section 3.5.1. One unit of coupling activity was defined as the amount of enzyme that can convert 1 μmol of β-CD/min.

### 3.6. Characterization of the Purified CGTase

#### 3.6.1. Estimation of the Molecular Weight of GTase

The molecular weight of the purified enzyme was determined by sodium dodecyl sulfate-polyacrylamide gel electrophoresis (SDS-PAGE), [43]. SDS-PAGE was performed on 7.5% polyacrylamide gel, using standard protein markers with molecular weights ranging from 10.5 to 175 kDa (Pink plus prestained protein ladder, GeneDirex). The non-specific staining of proteins with silver reagent in the polyacrylamide gel was carried out in order to detect minute concentration of proteins in the gel [44].

#### 3.6.2. Zymogram

For CGTase activity staining, the enzyme samples were applied to 10% native PAGE. After gel electrophoresis, the gel was washed twice with distilled water, and twice with 50 mM glycine-NaOH buffer, pH 8. Then, the gel was immersed in 50 mM glycine-NaOH buffer (pH 10) containing 1% (*w*/*v*) soluble starch, 0.02% (*w*/*v*) phenolphthalein and 1% (*w*/*v*) agar. After agar solidification, it was incubated for 1 h at 50 °C, and the CGTase activity was seen as a colorless band on a red background due to formation of stable colorless CD-phenolphthalein inclusion complex [14]. For detection of starch degrading activity, the native gel was immersed in solution of 1% (*w*/*v*) soluble starch in 50 mM Tris-HCl buffer (pH 8), and incubated for 1 h at 50 °C. Then, the starch solution was decanted and iodine solution was poured on the gel. The starch-degrading activity was seen as a clear band on dark blue background.

#### 3.6.3. Effect of Temperature on Activity and Thermostability of CGTase

The effect of temperature on the cyclization activity of CGTase was examined at various temperatures ranging from 35 °C to 70 °C under the standard assay conditions described above. The thermostability of the purified CGTse was determined by incubating the enzymes in 50 mM Trise-HCl (pH 8) at different temperatures (35 °C to 70 °C) and at various times interval (10–180 min), aliquots of enzyme were withdrawn and assayed for residual cyclization activity under the standard assay conditions. The thermal stability of the enzyme was further investigated in the presence of 1 mM calcium salt, 1% (*w*/*v*) of starch or maltodextrin, respectively, at varying temperatures from 50 °C to 70 °C for 1 h prior of determination of the residual enzyme activities under the standard assay conditions.

#### 3.6.4. Effect of pH on Activity Stability of CGTase

The effect of pH on the activity of CGTase was examined at various pH values ranging from pH 4.0 to 12.0, using suitable buffers including 50 mM sodium acetate (pH 5.0 and 6.0), 50 mM Trise-HCl (pH 7.0 and 8.0), 50 mM glycine-NaOH buffer (9.0 and 10.0) and 50 mM carbonate-bicarbonate buffer (11.0 and 12.0), respectively. In addition, the pH stability of purified enzyme was investigated by incubating the purified enzymes in buffers with different pH values for 1 h at room temperature, and the residual cyclization activities were assayed under the standard assay conditions. All experiments and enzyme assays were performed in triplicate and the mean values were reported.

#### 3.6.5. Effect of Metal Ions and Inhibitors on CGTase Activity

The effects of different metal ions and some inhibitors on the CGTase activity were investigated by addition of the test ions and reagents to reaction mixtures at final concentration of 1 mM or 10 mM. The test ions and reagents included Cu^2+^, Ca^2+^, Zn^2+^, Mn^2+^, Mg^2+^, Cd^2+^, Na^+^, Ba^2+^, Hg^2+^, Ni^2+^, Co^2+^, EDTA, 2-mercaptoethanol, α-, β-, and γ-CD.

#### 3.6.6. Kinetic Studies

Kinetic studies were performed by measuring the cyclization activity of CGTase at various concentrations of soluble starch ranging from 0% to 1.5 mg/mL. The kinetic constants, *K*_m_ and *V*_max_, were estimated using Michaelis-Menten equation and double reciprocal plot known as Lineweaver-Burk plot [33].

### 3.7. Cyclodextrin Production and Product Specificity of CGTase

The enzyme reaction was carried out at 50 °C, pH 8.0 using 1% (*w*/*v*) corn starch, and at defined time intervals the reaction was stopped by placing the samples in a boiling water bath for 5 min. The reducing sugars, in the reaction mixture, were measured as previously reported [31], and the starch consumption was followed by the method described by Krisman [45]. To eliminate contaminating oligosaccharides the starch hydrolysate were cooled and 50 μL was mixed with 5 μL (2 U) of glucoamylase and 45 μL 0.4 M sodium acetate buffer, pH 5.0, and incubated for 1 h at 40 °C. Then, the reaction was stopped by placing the samples in a boiling water bath for 5 min. The mixtures were filtered through a 0.45 μm membrane filter. The filtered samples (30 μL) were analysed by HPLC system using Aminex-HPX-42-A column (300 by 7.8 mm; Bio-Rad, Hercules, Calif.). CDs and linear sugars were eluted with degassed distilled water at flow rate of 0.6 mL/min. The flow cell was set at 80 °C and the products were detected by a refractive index detector (LaChrom, L7490 Merck-Hitachi, Ltd. Tokyo, Japan). Calibration curve was done using 1.0 mM, 2.5, 5.0, 7.5 and 10 mM of α-, β- and γ-cyclodextrin.

## 4. Conclusions

In this study, we report purification and characterization of a novel CGTase from *Amphibacillus* sp. NPST-10, isolated from hyper saline soda lakes. Enzyme purification to homogeneity was achieved by starch adsorption technique with enzyme yield of 44.7% and up to 22 fold purification. The purified enzyme was found to be a monomeric protein with an estimated molecular weight of 92 kDa. The applied purification procedure is easily feasible under industrial conditions. In comparison to other CGTases obtained from alkaliphiles, CGTase from *Amphibacillus* sp. NPST-10 could be effectively used for conversion of raw starch into cyclodextrins in a wide pH range, from 6.0 to 11.0 and temperatures ranging from 45 °C to 65 °C. Moreover, the enzyme exhibited a good thermostability being stable for at least 1 h at 30 °C to 55 °C. *Amphibacillus* sp. NPST-10 CGTase was effectively active on raw corn starch with high conversion rate (67.2%) with predominant formation of β-CD (86.4%), making this enzyme favorable for industrial application. Further work on cloning of *cgtase* gene from *Amphibacillus* sp. NPST-10 and analysis of CDs production by immobilized enzyme are in progress.

## Figures and Tables

**Table 1:** Purification of CGTase from *Amphibacillus* sp. NPST-10.

Purification step	Volume(mL)	Activity(U mL^−1^)	Protein(mg mL^−1^)	Specific activity (U mg^−1^)	Yield(%)	Purification (Fold)
Crude	1000	379.0	94.5	4.0	100	1.0
Starch adsorption eluate	285	177.5	2.0	88.8	44.7	22.2
DEAE-Cellulose fractions	8	144.2	1.6	89.0	36.3	23.1

**Table 2:** Effect of various ions and reagents on the activity of *Amphibacillus* sp. NPST-10 CGTase. ND: Not determined.

Salts/Reagent	Residual Activity (%)	

	1 mM	10 mM
CuCl_2_	99.2 ± 1.0	32.8 ± 1.5
CaCl_2_	101.0 ± 2.0	108.7 ± 1.9
ZnCl_2_	89.1 ± 1.9	40.3± 2.5
MnSO_4_	93.1 ± 0.9	88.0 ± 0.8
MgCl_2_	90.9 ± 0.85	96.6 ± 0.7
CdCl_2_	82.6 ± 1.1	29.3 ± 1.0
NaSo_4_	96.0 ± 0.9	94.7 ± 0.7
BaCl_2_	85.1 ± 2.1	65.7 ± 1.9
HgBr_2_	85.1 ± 2.0	0.0
NiCl2	98.1 ± 0.8	101.2 ± 0.5
Co(NO_3_)_2_	100.9 ± 1.0	82.1 ± 1.2
EDTA	100.8 ± 0.7	99.5 ± 0.6
2-ME	98.8 ± 0.6	76.7 ± 0.7
α-CD	68.7 ± 2.3	N/A
β-CD	45.7 ± 2.2	N/A
γ-CD	54.2 ± 1.8	N/A
